# Clinical-Pathological Conference Series from the Medical University of Graz: Case No 153: A 55-year-old woman with atypical multiple sclerosis and irritable bowel syndrome

**DOI:** 10.1007/s00508-017-1291-y

**Published:** 2017-11-21

**Authors:** Elisabeth Fabian, Dietmar Schiller, Hermann Toplak, Michaela Brunner-Krainz, Franz Fazekas, Rainer Schoefl, Guenter J. Krejs

**Affiliations:** 1 Division of Gastroenterology and Hepatology, Department of Internal Medicine III, Medical University of Vienna, Vienna, Austria; 2 Department of Medicine IV, Elisabethinen Hospital, Linz, Austria; 3 Department of Internal Medicine, Medical University of Graz, Graz, Austria; 4 Department of Pediatrics, Medical University of Graz, Graz, Austria; 5 Department of Neurology, Medical University of Graz, Graz, Austria; 6 Division of Gastroenterology and Hepatology, Department of Internal Medicine, Medical University of Graz, Auenbruggerplatz 15, 8036 Graz, Austria

**Keywords:** Fabry disease, Atypical multiple sclerosis, Irritable bowel syndrome

## Presentation of case

### Dr. D. Schiller:

A 55-year-old retired female secretary was admitted to hospital due to vague abdominal pain and diarrhea (three mushy stools per day) during the last 8 months. She had neither fever nor reduced appetite and had not lost weight. She is married, has two healthy children, has never travelled outside Europe and does not smoke or drink alcohol. Her medical history includes repeated episodes of neurological symptoms, including transitory paresis of the left arm, vertigo and disturbed equilibrium. Once she also experienced transient paresthesia of the left leg. Magnetic resonance imaging (MRI) of the head during the last 10 years revealed slightly progressing white matter lesions. Cerebrospinal fluid (CSF) showed 50 cells/µl (85% lymphocytes, 15% monocytes) and increased concentration of protein (75 mg/dl, normal: 15–40 mg/dl), but without oligoclonal bands. Based on these findings, atypical multiple sclerosis (MS) was diagnosed. Therapy with glucocorticoids for 9 months had shown a temporary positive effect, but a therapeutic trial with interferon-beta 1a was futile.

For many years the patient suffered from headaches, arthralgia, diffuse myalgia and fatigue without signs of inflammation. Several neurological and rheumatological consultations failed to provide a clear diagnosis. At the age of 49 years the patient took premature disability retirement.

Arterial hypertension had been treated with lisinopril (5 mg per day) for the last 7 years. The dosage of lisinopril was increased to 10 mg per day 2 months before admission because the 24 h ambulatory blood pressure measurement gave a mean pressure of 150/95 mm Hg. The patient did not take any other medication.

On admission physical examination was without pathological findings except for pain on deep abdominal palpation but there was no guarding or rebound phenomenon. There was no hepatomegaly or splenomegaly. Skin and mucous membranes were unremarkable. Results were normal or negative for extensive laboratory tests, stool cultures, fecal calprotectin, abdominal ultrasonography, chest and abdominal computed tomography (CT), esophagogastroduodenoscopy and colonoscopy with biopsies, and MRI of the small bowel and the visceral vasculature performed 2 months earlier.

Selected laboratory results: erythrocyte sedimentation rate (ESR) within the first hour was 22 mm (normal: <20 mm), C‑reactive protein (CRP) 0.7 mg/dl (normal: <0.5 mg/dl); all the other parameters were normal or negative including cholesterol, triglycerides, thyroid stimulating hormone, free thyroxine, immunoglobulins (Ig)G, IgA, IgM, anti-tissue transglutaminase, serological tests for several viruses and borrelia, human immunodeficiency virus (HIV), *Treponema pallidum* hemagglutination assay (TPHA), and cytomegalovirus (CMV) PCR. Serological tests for several autoimmune antibodies were also negative, as were the results of urinalysis and microalbumin. Ultrasonography of the carotid arteries was negative for atherosclerosis and the echocardiogram identified hypertensive cardiomyopathy (thickness of septum 15 mm).

An important finding leading to the final diagnosis was provided by a simple, noninvasive examination.

## Differential diagnosis

### Dr. H. Toplak:

The patient under discussion is a 55-year-old woman who was admitted to hospital due to atypical MS and irritable bowel syndrome (IBS). Focusing on the term atypical MS and considering the medical history of the patient, one gets the impression that this diagnosis is more speculative than definitive, although it could point to another diagnosis, such as a metabolic disorder. The notion of IBS is also not very helpful for finding the final diagnosis, because it is a diagnosis of exclusion. Taken together, these are two vague diagnoses that according to the protocol will lead to a final diagnosis on the basis of a simple, noninvasive test.

Although the patient did not report weight loss, malabsorption syndromes have to be considered. Celiac disease was excluded by duodenal biopsy and serology. Whipple’s disease can be excluded because it is associated with progressive weight loss, abdominal pain, lymph node involvement, enteropathic arthritis, spondyloarthropathy, microcytic hypochromic anemia, and further signs and symptoms not observed in this case. Moreover, the neurological symptoms in Whipple’s disease are not transient as described for this patient and the disease is very rare in women.

The patient reported a 15-year history of neurological symptoms, such as transient paresis of the left arm, vertigo and disturbance of equilibrium, transient paresthesia and headaches. The MRI showed slightly progressive white matter lesions over the past 10 years, resulting in the diagnosis of atypical MS. White matter lesions can, however, also occur in systemic diseases, such as systemic lupus erythematosus, scleroderma, Sjögren’s syndrome and idiopathic demyelinating diseases. Since several autoimmunological parameters were negative and CSF analysis did not show oligoclonal bands, i. e. there was no increase of Igs in CSF, an autoimmune disease can most likely be excluded. Laboratory blood and CSF data, however, revealed mild chronic inflammation, probably due to an infection or a reaction to something as yet unknown. Viral infection with Epstein-Barr virus (EBV), CMV and tick-borne viral meningoencephalitis also causes white matter lesions, as does bacterial infection with *Borrelia* (neuroborreliosis), but serological tests for several viruses and *Borrelia* were negative. Other tests for evaluation of any viral or bacterial infection could possibly have been invasive and so are ruled out because the final diagnosis was made by performing a simple, noninvasive test.

The information that a therapeutic trial with glucocorticoids for 9 months had a temporary positive effect but administration of interferon-beta 1a did not improve symptoms, is not really helpful for reaching a final diagnosis because we have no details on dosage and definite duration of the therapies. Information about certain neurological symptoms persisting for years, arterial hypertension and physical examination without abnormal abdominal findings except for abdominal discomfort on deep palpation does also not really help to establish a final diagnosis. From all the results so far, only the finding of hypertensive cardiomyopathy (thickness of septum 15 mm) seems to be important for the further differential diagnosis.

Due to the involvement of multiple organ systems with no obvious morphological correlation and the thickened cardiac septum, chronic intoxication could be suspected to cause this woman’s health problems. Chronic lead poisoning can for instance result in MS-like symptoms or cause migraine and fatigue. Chronic exposure to amalgam containing mercury, cadmium and aluminum could also cause such symptoms. Moreover, vasculitis can induce white matter lesions and cause abdominal pain. Side effects of chemotherapy have been reported to be similar to the symptoms seen here, but this can be ruled out due to the negative history. Further diagnoses, such as amyloidosis, chronic fatigue syndrome, hypothyroidism and disturbances of the pituitary/adrenal axis may show some similar symptoms but do not seem to suffice to explain this patient’s problems.

Taken together there are four important features for the establishment of the final diagnosis: (1) probable chronic intoxication with reactive inflammation, (2) a thickened cardiac septum, (3) white matter lesions and (4) IBS.

The family history of the patient was reported to be negative. Actually, it is so unremarkable that it should be checked again in more detail. In daily routine physicians always focus on well-defined diseases with characteristic symptoms. In children with unclear or nonspecific symptoms, genetically determined metabolic diseases are more likely to be considered than in adults. Various metabolic diseases can cause severe symptoms but depending on the genotype may also present with milder symptoms. In individuals with mild genetic defects or in those who are heterozygous, especially X‑linked heterozygous, symptoms are often discrete rather than severe and distinct. Adrenoleucodystrophy (ADL) and adrenomyeloneuropathy (AMN), for example, are X‑linked inherited metabolic diseases due to disturbed peroxisomal degradation of very long chain fatty acids. Depending on the ADL genotype, the phenotype significantly differs depending on the age at which the disease is manifested and the symptoms, which vary from mild to very severe. Keeping this in mind, I would strongly suggest that the discussed patient suffers from a mild form of a certain metabolic disorder.

Considering all the available information, I think that she suffers from a lysosomal storage disease, since these disorders are known to cause the typical neurological symptoms observed in this case [1]. Absent or deficient activity of lysosomal exoglycohydrolase α‑galactosidase A (α-D-galactoside galactohydrolase, EC 3.2.1.22) [2, 3] results in progressive accumulation of globotriaosylceramide (Gb_3_, GL-3 or ceramidetrihexoside [CTH]) and related glycosphingolipids (galabiosylceramide) within lysosomes [4] of various cell types including capillary endothelial cells, renal (podocytes, tubular cells, glomerular endothelial, mesangial and interstitial cells), cardiac (cardiomyocytes and fibroblasts) and nerve cells [5, 6]. Depending on the affected cells and the degree of involvement at different sites, symptoms will vary from patient to patient. Since Gb_3_ can be classified as a kind of neurotoxin, it also causes specific neurological symptoms, as seen in the discussed patient. This progressive, X‑linked inherited disorder of the lysosomal glycosphingolipid metabolism was first but independently described in 1898 by Johannes Fabry and William Anderson [7, 8] and is thus known as Fabry disease or Anderson-Fabry disease. Both physicians observed a specific distribution of angiokeratoma corporis diffusum in their patients that is characteristic for Fabry disease. Since Fabry disease is an X‑linked inherited disease, classically hemizygous males with no residual α‑galactosidase A activity may display all the characteristic neurological, cutaneous (angiokeratoma), renal (proteinuria, kidney failure), cardiovascular (cardiomyopathy, arrhythmia), cochlear-vestibular and cerebrovascular (transient ischemic attacks, strokes) symptoms of the disease, while heterozygous females often have mild symptoms [6]. In the past, female heterozygotes were erroneously described as “carriers of the defective gene” and thought to be safeguarded against developing signs and symptoms of the disease. However, women inherit one X chromosome from each parent; in each cell, one X chromosome is randomly inactivated while the other one is active and provides the genetic information. According to the Lyon hypothesis, the degree of X inactivation will determine whether females have a favorable or unfavorable phenotype [1]. This suggests that it is more appropriate to describe Fabry disease with a wide spectrum of manifestations that range from the classical phenotype in males to a seemingly asymptomatic disease course occasionally observed in females, with a variety of clinical presentations in between. Most female heterozygotes develop symptoms due to yet undetermined mechanisms [9–11]. Females often show vital organ involvement including kidneys, heart and brain approximately a decade later than males [9]. With age, progressive damage to vital organ systems resulting in organ failure develops in both genders [9]. End-stage renal disease and life-threatening cardiovascular or cerebrovascular complications reduce life expectancy [12–15]. Fabry disease affects multiple organ systems; early signs and symptoms of the disease are summarized in Table 1. Neurological symptoms are present in almost every patient, cutaneous and renal symptoms are seen in every second patient. Half of the patients also show the characteristic cornea verticillata [16] that rarely affects vision but is readily detectable by slit lamp examination, i. e. “a simple, noninvasive examination that led to the final diagnosis” as stated in the protocol. Posterior subcapsular spoke-like cataracts and retinal vessel tortuosity are also seen [17].

**Table 1:** Early signs and symptoms of Fabry disease [6]

Organ system	Sign/symptom
Nervous system	Acroparesthesia
	Nerve deafness
	Heat intolerance
	Hearing loss, tinnitus
Gastrointestinal tract	Nausea, vomiting, diarrhea, postprandial bloating and pain, early satiety
	Difficulty gaining weight
Skin	Angiokeratomas
	Hypohidrosis
Eyes	Corneal and lenticular opacities
	Vasculopathy (retina, conjunctiva)
Kidneys	Microalbuminuria, proteinuria, impaired concentration, hyperfiltration, increased urinary Gb_3_ excretion
Heart	Heart rate variability, arrhythmias, abnormal electrocardiogram (shortened PR interval), mild valvular insufficiency

Cardiac symptoms, such as left ventricular hypertrophy, arrhythmia, angina and dyspnea are reported in about 40–60% of patients with Fabry disease [14, 18–21]. These patients often have left ventricular abnormalities. The septum thickness in particular can show significant alterations since the posterior wall may become thinner with age due to fibrosis; concentric hypertrophy has been reported as the most common structural change [18] and was also seen in our patient. The cardiomyopathy in Fabry disease is characterized by reduced myocardial contraction and relaxation. Right ventricular hypertrophy with normal chamber size and preserved systolic but impaired diastolic function reflects the typical right ventricular structural and functional changes in this disease [6]. Right ventricular wall thickness, age and left ventricular mass index are significantly correlated in Fabry disease [22] and there is a relationship between the degree of right ventricular involvement and the left ventricular cardiomyopathy stage [23].

Gastrointestinal symptoms have been reported in 50–70% of patients with Fabry disease [12, 13] and frequently include diarrhea and abdominal pain [24]. These symptoms may be due to the deposition of Gb_3_ in the enteric ganglia and mesenteric blood vessels [25]. Many patients have an alternating pattern of diarrhea, normal stool or constipation. This clinical manifestation is reminiscent of IBS, as are symptoms of abdominal discomfort and bloating associated with food intake [26]. Patients with Fabry disease tend to be diagnosed with diarrhea-predominant IBS [27].

Due to the described symptoms, patients with Fabry disease have significantly lower quality of life than the healthy population [28], comparable to that of patients with AIDS and even worse than the quality of life reported by patients with Gaucher disease [29]. Approximately 18% of patients suffer from psychiatric complaints including depression, often leading to suicide [30, 31].

## Dr. H. Toplak’s diagnosis

Fabry disease

### Discussion of diagnosis

#### Dr. D. Schiller:

In this interesting case there are two important hints for making the final diagnosis. First, the discrepancy of mild hypertension and an increased septum thickness (15 mm) and secondly, the medical history of different unclear neurological symptoms suggesting MS or repeated cerebrovascular insults. Due to these symptoms and former diagnoses, an ophthalmologist was consulted and detected cornea verticillata by slit lamp examination. Cornea verticillata, first described by Fleischer in 1901, is a whorl-shaped dystrophy of the cornea characterized by a fine stippling of the epithelium and Bowman’s membrane. The opaque brownish stipples are arranged in curved lines that converge toward an inferior internal paracentral point. This important finding finally led to the diagnosis of Fabry disease. Patients with the disease frequently have cornea verticillata due to accumulation of Gb_3_ in Bowman’s membrane. Therapeutic options include the administration of amiodarone or chloroquine. After Gaucher disease, Fabry disease is the second most frequently observed disorder of lysosomal glycosphingolipid metabolism. As Dr. Toplak already said, Fabry disease is an X‑linked lysosomal storage disease defined by the absence or deficiency of α‑galactosidase, leading to progressive accumulation of Gb_3_ and related glycosphingolipids in lysosomes. Lysosomal α‑galactosidase A is coded by a unique gene (GLA) located on the long arm of chromosome X (Xq22). This gene consists of 7 exons distributed over 12,436 bp. Fabry disease can be due to a variety of missense or nonsense point mutations, splicing mutations, small deletions or insertions, and large deletions [6]. Defects in the gene are heterogeneous with over 700 mutations recorded [32, 33]. Genotyping of our patient revealed a heterozygote deletion in exon 7 (c.1124_1129del p.G375_V376del). Biochemically, we observed a significantly increased plasma concentration of Gb_3_ (9.01 ng/ml, normal: ≤2.7 ng/ml). Due to the genetic background of this disease resulting in a variety of manifestations, genetic analysis should always be done to confirm the diagnosis.

In Fabry disease different organ systems, such as the nervous system, eyes, ears, heart, kidney, gastrointestinal tract and skin can be involved. Neurological and gastrointestinal symptoms were the most prevalent clinical features in our patient. As in her case, many heterozygous patients with Fabry disease first show neurological symptoms in their mid-30s. In addition to the neurological symptoms already described by Dr. Toplak, this disease may also manifest with chronic meningitis [34]. Nonspecific neurological symptoms are often important presenting features of the disease, but can be misdiagnosed for many years as e. g. MS, as in this case and thus delay proper treatment for Fabry disease. Moreover, I would also like to point out that Fabry disease may be underestimated in the differential diagnosis of MS, probably leading to misdiagnosis or delayed diagnosis and treatment [35].

As far as gastrointestinal symptoms are concerned, IBS type diarrhea is very common in Fabry disease (52%), with children most frequently affected followed by women and men [27]. Thus, in patients with unclear or nonspecific gastrointestinal symptoms, Fabry disease should also be considered to avoid misdiagnosis as reported by Zizzo et al. [36].

As the main treatment of Fabry disease, enzyme replacement therapy is available with preparations such as agalidase alpha (Replagal®, Shire, Cambridge, MA, USA, 0.2 mg/kg i. v. biweekly) and agalidase beta (Fabrazyme®; Genzyme Corp, Cambridge, MA, USA; 1.0 mg/kg i. v. biweekly). This therapy has been shown to stabilize and possibly reverse symptoms [37].

Due to the variety of phenotypes, I would like to end my comments by comparing Fabry disease to a chameleon that can hang out in practices and clinics for years, i. e. it can take a very long time to arrive at a clear diagnosis and provide effective treatment.

#### Dr. G.J. Krejs:

Gastrointestinal symptoms are some of the most frequent and early complaints in patients with Fabry disease, affecting up to 70% of patients [38]. The symptoms occur anywhere along the gastrointestinal tract, vary in intensity and frequency, and include a wide range of clinical features, such as abdominal pain, delayed gastric emptying and early satiety, nausea, bloating, diarrhea and constipation. Patients may experience one severe symptom or a combination of symptoms [37]. Gastrointestinal symptoms in Fabry disease are thought to be due to Gb_3_ accumulation, leading to neuronal and vascular dysfunction, disruption of cellular signaling and subsequently to ischemia, inflammation and malfunction [39]. Abdominal pain is the most common complaint whereby diarrhea is the second most common gastrointestinal symptom, occurring in 20% of Fabry patients, and is associated with significant urgency (sometimes fecal incontinence) and frequency (up to 15 bowel movements per day). Patients with Fabry disease do not have blood or mucus in the stool [27]. The clinical course of gastrointestinal symptoms in Fabry disease is often similar to that encountered in various other disease presentations and so may lead to delayed diagnosis and mistreatment [37]. Recent data revealed that 64% of adults and 25% of children with Fabry disease also meet the criteria for a functional gastrointestinal disorder, making the diagnosis even more difficult [40].

Dr. Brunner-Krainz is a pediatrician experienced in treating patients with inborn errors of metabolism and will now talk about the diagnosis of Fabry disease.

#### Dr. M. Brunner-Krainz:

As mentioned before, Fabry disease belongs to a group of lysosomal storage disorders (LSDs), which includes diseases with different underlying etiologies: (1) disturbances in the metabolism of complex carbohydrates (e. g. mucopolysaccharidosis, α‑mannidosis, α‑fructosidosis, Pompe disease), (2) defects of membrane proteins (e. g. mucolipidosis II, III, IV, Salla disease), (3) defects of lysosomal enzymes or transport and activator proteins (sphingolipidoses: Gaucher disease, Niemann-Pick disease, Fabry disease, Faber disease, Krabbe disease) and (4) defects in degradation of gangliosides (GM I, GM II gangliosidosis, Tay Sachs disease, Sandhoff disease). Each disorder is caused by a monogenetic defect resulting in deficiency or absence of lysosomal enzyme(s). Therapeutic options include hematopoietic stem cell therapy, gene therapy (e. g. virus vector therapy) and enzyme replacement therapy (ERT).

The incidence of Fabry disease is reported to range from 1 in 40,000 to 1 in 476,000 in the general population [6, 41, 42]. Pilot programs of newborn screening (NBS) found a prevalence ranging from 1 in 1500 to 1 in 3100 [43, 44]. This prevalence should, however, be viewed critically because it includes females with unpredictable disease course and males with non-classical disease. Furthermore, NBS data raise many questions: (1) Should ERT be started immediately after the initial diagnosis in NBS or should this therapy commence after the first clinical symptoms develop? (2) How frequently should follow-up assessments be performed? (3) What burden on the families and costs does lifelong ERT entail? Today, in Austria Fabry disease is only diagnosed in symptomatic patients and not by NBS. For family members of index patients, early diagnosis, prenatal diagnosis and genetic counselling are available.

The correct diagnosis of Fabry disease is established by analysis of lysosomal α‑galactosidase A activity in leukocytes or dried blood spot and by genetic testing. Biochemical markers, such as Gb_3_ and lyso-Gb_3_ could help in the diagnosis and in the follow-up of treated patients. However, they can also cause some problems because Gb_3_ is generally lower in females than in males and is often within the normal range in females with Fabry disease [45]. Lyso-Gb_3_ is a water-soluble substance and is elevated in hemizygous males and to a lesser extent in females with classical Fabry disease, but in general, plasma concentrations of lyso-Gb_3_ correlate with the overall disease severity of patients [46–48]. Moreover, lyso-Gb_3_ is suggested as a marker to monitor ERT [49]. Aerts et al. [46] found that under ERT, concentrations of Gb_3_ decrease while Gb_3_ metabolites stay high, suggesting further unknown metabolites and rendering the therapeutic effect of ERT questionable. Plasma lyso-Gb_3_ concentration was found to correlate with white matter lesions and left ventricular mass, and is correlated with clinical manifestations in patients with Fabry disease [48]. In summary, the diagnostic value of plasma lyso-Gb_3_ is still under debate [50]. Urinary lyso-Gb_3_ may also be a potential biomarker [49]; there are, however, two mutations that do not result in formation of lyso-Gb_3_ [46]. Lyso-Gb_3_ is not detectable in healthy subjects [46].

As explained by Dr. Schiller, the GLA gene is located on chromosome X and consists of 7 exons distributed over 12,436 bp. Currently, a total of 751 different mutations of the GLA gene have been reported in the Human Gene Mutation Database [33]. Depending on the genotype the mutations can be classified as pathogenic, probable pathogenic or apathogenic (i. e. the mutation causes reduced but normal range enzyme activity). According to the Human Gene Mutation Database, only a few mutations cause an atypical course of the disease [33].

ERT has been available for Fabry patients since 2001 and two preparations are on the market: Replagal® and Fabrazyme® as mentioned by Dr. Schiller. Another new therapeutic option is offered by active site-specific chaperones (iminosugars). In Fabry disease many disease-causing mutations are missense mutations leading to unstable but still catalytically stable lysosomal protein [51, 52]. Due to this unstable conformation, enzymes are unable to undergo trafficking to their appropriate location within the cell; mutated enzymes are retained in the endoplasmic reticulum and degraded because of their misfolded conformation [53]. Active site-specific chaperones assist protein folding and stabilize misfolded proteins [54]. Amigal® (migalastat hydrochloride, Amicus Therapeutics, Cranbury, NJ, USA) is a preparation that binds to the mutant enzyme, shifting the folding and stability of the enzyme in favor of the correct conformation, potentially permitting a smooth escape from the endoplasmic reticulum for further maturation and trafficking to the lysosomal compartment [55]. This therapy, however, can only be provided to patients with specific responsive GLA mutations coding for a mutant α‑galactosidase A with enhanceable residual enzyme activity [6].

#### Dr. G.J. Krejs:

The patient was diagnosed with atypical MS and also had repeated MRIs. Dr. Fazekas will comment on the MRIs and report on his experience with Fabry disease as a neurologist.

#### Dr. F. Fazekas:

**Fig. 1:**
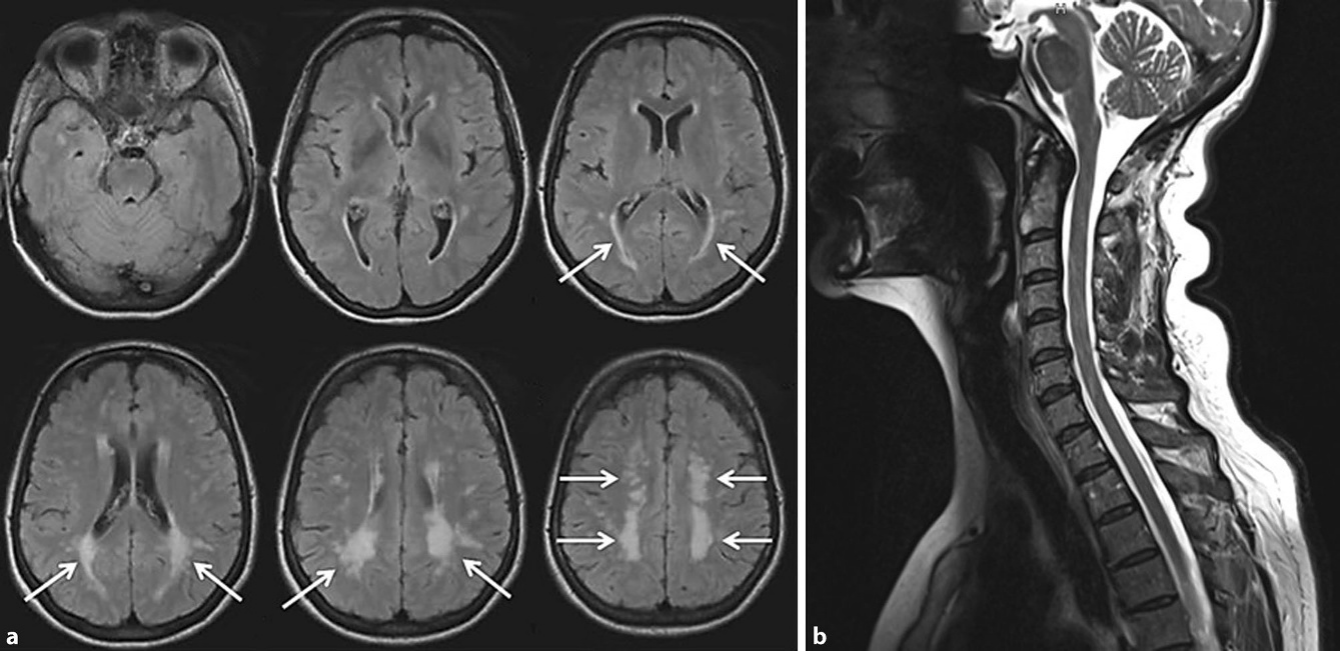
Magnetic resonance images of the brain (**a**) and spinal cord (**b**). **a** shows six axial FLAIR sections through the brain in craniocaudal direction starting at the brainstem. Extensive and rather symmetric white matter changes (*arrows*) located preferentially in both parietal lobes and along the lateral ventricles are seen. The subcortical U-fibres and infratentorial brain parenchyma are spared. **b** is a sagittal-T2-weighted section of the cervical and upper thoracic spine. There are some artifacts but no definite lesion is seen in the spinal cord. *FLAIR* fluid-attenuated inversion recovery

First of all I have to say that the term atypical MS is no longer used; today we speak of possible or definite MS. The use of MRI plays an important role in diagnosing MS as only 1 in 5–10 MS lesions becomes clinically apparent. Unfortunately, MS lesions on MRI can only be suspected because of their distinct location while the signal characteristics are nonspecific and similar to white matter hyperintensities of several etiologies including Fabry disease. Confusion between MS and Fabry related MRI lesions is not uncommon if MRI findings are interpreted liberally [35]. Accordingly, it is important to first consider whether the clinical findings are at all suggestive of MS [56]. The age of 38 years when the first neurological symptoms occurred with repeated deficits including transitory paralysis of the left arm, vertigo and disturbance of equilibrium, and transitory paresthesia could certainly be compatible with MS. For a diagnosis of MS, however, symptoms have to last for at least 24–48 h, but unfortunately, the duration of transitory paralysis and paraesthesia was not reported in this case. Results of the CSF analysis were compatible with but not suggestive of MS. The CSF of MS patients is characterized by mild pleocytosis (<25 cells/µl), slightly increased protein content and most specifically by the presence of oligoclonal bands that are found in 85–95% of affected subjects but were not seen in this patient. The MRI (Fig. 1) showed extensive white matter hyperintensities in a rather symmetric distribution with a preference for the periventricular and deep parietal white matter, sparing the subcortical U‑fibers. There were no lesions in the brainstem or cerebellum and the spinal cord also did not show any of the lesions that are present in about 80% of patients with established MS, even in the absence of spinal cord symptoms [56]. Diagnostic criteria for MS require the presence of one or more T2 lesions in at least 2 out of 4 areas of the CNS (periventricular, juxtacortical, infratentorial, spinal cord) and the accumulation of new lesions over time [57], but this patient did not provide any such proof for dissemination in space and time.

It can be speculated that the patient’s neurological symptoms were of vascular origin, although the MRI did not show a clear infarct. The prevalence of stroke, which can be the first manifestation of Fabry disease, is estimated to be 7% in men and 4% in women [58]. Data from the Fabry Registry® [58] and the Fabry Outcome Survey® [59] show that the majority of strokes in Fabry disease are due to small vessel events. In a multinational European study of 5023 individuals with ischemic or hemorrhagic stroke or transient ischemic attacks (SIFAP 1), evidence for definite Fabry disease was present in 27 (0.5%) and for probable Fabry disease in an additional 18 (0.4%) patients [60]. White matter lesions may be single, patchy or confluent on MRI. Additionally, T1 high-signal intensity of the pulvinar thalami and tortuous, ectatic blood vessels characterize MRI in Fabry disease but are not specific for it [61]. In a blinded MRI interpretation of 3203 patients in the SIFAP 1 study, none of the morphological changes of the brain reported in Fabry disease could differentiate such patients from patients with stroke or transient ischemic attacks due to other causes [62]. Pulvinar hyperintensity was not seen in any of the Fabry patients in this series but in six patients without Fabry disease; therefore, MRI of the brain cannot be used as a screening tool for Fabry disease and suspicious MRI changes always have to be viewed in the light of findings for other organ systems and appropriate laboratory and genetic work-up to establish the final diagnosis.

#### Dr. D. Schiller:

Unfortunately, it is quite difficult to get a precise medical history from a patient who has had nonspecific symptoms for 15 years. The patient could not remember all of the details, but recalled that she had had transient paralysis of the left arm 3 times, lasting about 1 min, and repeated paresthesia that lasted approximately 36 h; the durtion of the latter being compatible with MS. She is currently under ERT.

#### Dr. H. Pristautz:

One critical remark: I think we have to question whether ERT is a good therapeutic option for adults with Fabry disease. Patients have a long medical history, a wide variety of symptoms and variable disease progression and since laboratory markers hardly help, it is difficult to prove or document a therapeutic benefit of ERT. Does ERT given to children prolong life expectancy and who pays for ERT?

#### Dr. M. Brunner-Krainz:

Treatment guidelines have been available since 2009. In a revised version in 2015 [63] ERT recommendations were based on clinical experience, observational studies and randomized controlled trials using recommendation classes I (best evidence), II, (IIA, IIB) and III. Class I recommendations apply to patients with proteinuria and cardiac hypertrophy or cardiac arrhythmias. In patients with white matter lesions ERT may be considered (class IIB). In patients with acroparesthesia or IBS ERT may be considered (class IIA, IIB).

The benefit of ERT is well documented in registries (i. e. Fabry Outcome Survey®) but there are still patients with rapid disease progression despite ERT. The role of inhibitory antibodies to ERT is still not clear. In Austria, payment for this therapy varies among the states. Usually, costs are covered by the health insurance provider or by the hospital.

#### Dr. G.J. Krejs:

In 153 clinical-pathological conferences in almost 30 years we have never had a case of Fabry disease. Today Fabry disease was compared to a chameleon but there are also other medical chameleons that we have discussed here, such as Wegener’s disease, Whipple’s disease and sarcoidosis that can present with a wide variety of symptoms and affect different organ systems. What we learned from this case is to be more aware of rare diseases that can cause such common problems as IBS.

#### Dr. H. Toplak:

Several years ago I worked on lysosomal metabolic disorders but I have never seen a patient with Fabry disease. My final remark focuses on a certain group of medications that is widely used in patients with different diseases. Lysosomotropic medications, such as tricyclics like desipramine [64] as well as the antimalarial drug chloroquine [65] and the antiarrhythmic amiodarone [66] are stored in the lysosomes and can cause secondary reductions in the activity of lysosomal enzymes. This may cause problems in patients with latent metabolic disturbances and result in secondary storage diseases.

## Final diagnosis

Fabry disease
